# Identification of candidate aberrantly methylated and differentially expressed genes in thyroid cancer

**DOI:** 10.1002/jcb.27129

**Published:** 2018-08-02

**Authors:** Yaqin Tu, Guorun Fan, Hongli Xi, Tianshu Zeng, Haiying Sun, Xiong Cai, Wen Kong

**Affiliations:** ^1^ Department of Otorhinolaryngology Union Hospital, Tongji Medical College, Huazhong University of Science and Technology Wuhan 430022 China; ^2^ Department of Hepatobiliary Surgery Union Hospital Tongji Medical College Huazhong University of Science and Technology Wuhan 430022 China; ^3^ Department of Clinical laboratory Cancer Center of Guangzhou Medical University Guangzhou China; ^4^ Department of Endocrinology Union Hospital, Tongji Medical College, Huazhong University of Science and Technology Wuhan 430022 China

**Keywords:** bioinformatical analysis, hub genes, methylation, pathways, thyroid cancer (THCA)

## Abstract

Aberrant methylation of DNA sequences plays a criticle role in finding novel aberrantly methylated genes and pathways in thyroid cancer (THCA). This study aimed to integrate three cohorts profile datasets to find novel aberrantly methylated genes and pathways in THCA. Data of gene expression profiling microarrays (GSE33630 and GSE65144) and gene methylation profiling microarrays (GSE51090) were downloaded from the Gene Expression Omnibus database. Aberrantly methylated and differentially expressed genes were sorted and pathways were analyzed. Functional and enrichment analyses of selected genes were performed using the String database. A protein‐protein interaction network was constructed using the Cytoscape software, and module analysis was performed using Molecular Complex detection. In total, we identified 12 hypomethylation/high‐expression genes and 30 hypermethylation/low‐expression genes at the screening step and, finally, found 6 mostly changed hub genes including *PPARGC1A*, *CREBBP*, *EP300*, *CD44*, *SPP1,* and *MMP9*. Pathway analysis showed that aberrantly methylated differentially expressed genes were mainly associated with the thyroid hormone signaling pathway, AMP‐activated protein kinase (AMPK) signaling pathway, and cell cycle process in THCA. After validation in the Cancer Genome Atlas database, the methylation and expression status of hub genes was significantly altered and the same with our results. Taken together, we identified novel aberrantly methylated genes and pathways in THCA, which could improve our understanding of the cause and underlying molecular events, and these candidate genes could serve as aberrant methylation‐based biomarkers for precise diagnosis and treatment of THCA.

## 1 INTRODUCTION

Thyroid cancer (THCA) is a common endocrine malignancy. Globally, the incidence of THCA has risen rapidly in recent decades, and the prevalence accounts for approximately 5.11% of malignant head and neck tumors. The average annual increase in the incidence of THCA is nearly 6.6%, which is the highest among all cancers.^1^ THCA affects women more often than men (female‐to‐male ratio 1:2 to 1:3), and it usually occurs in people aged between 25 and 65 years.^2^ Multiple factors might cause THCA, including obesity, smoking, overweight, and radiation exposure.^3^ The accumulation of various genetic and epigenetic alternations in thyroid follicular epithelial cells is also regarded as an essential process in driving the initiation and progression of THCA.^4^, ^5^

As we know, epigenetics involves heritable alterations in gene expression that are not mediated by changes in the DNA sequence. DNA methylation is closely related to embryonic development,^6^ regulation of gene expression,^7^ X‐chromosome inactivation,^8^ genomic imprinting,^9^ and genomic stability.^10^ Aberrant methylation of DNA sequences, including hypermethylation of tumor‐suppressor genes and hypomethylation of oncogenes, has been implicated to play a critical role in tumorigenesis. Amongst others, *P16*,^11^ *Ras association domain family 1 isoform A*,^11^ *mammary serpin*,^12^ and *CDKN2/p16* ^*INK4A*^ 13 are inactivated in THCA due to hypermethylation in the promoter region of the genes. Considering that there are no typical symptoms in the early stages of thyroid neoplasia, early detection of THCA by genetic diagnosis is crucial to guide the treatment and to improve the prognosis of THCA.

Owing to the application of a high‐through sequencing method, the identification of disease‐related biomarkers is effectually stated in many research articles.^14^, ^15^, ^16^, ^17^, ^18^, ^19^, ^20^ Gene expression profiling has been used to find various differentially expressed genes (DEGs), and DNA methylome analysis has made it possible to identify differentially methylated genes (DMGs). Although multiple studies have demonstrated specific genes with aberrant DNA hypermethylation or hypomethylation in THCA,^21^, ^22^ the comprehensive profile and pathways of the interaction network remain elusive.

The comprehensive analysis of multiple datasets possesses sufficient power to identify key genes and pathways involved in cellular processes and biological functions compared with individual investigations. In the current study, data of gene expression profiling microarrays (GSE33630 and GSE65144) and gene methylation profiling microarrays (GSE51090) were integrated and analyzed by a series of bioinformatics tools. Aberrantly methylated DEGs and pathways were identified in THCA. A protein‐protein interaction (PPI) network was constructed, and hub genes were revealed. By this means, we expect to find novel aberrantly methylated genes and pathways in THCA, and shed light on the underlying molecular mechanisms that orchestrate thyroid carcinogenesis.

## 2 METHODS

### 2.1 Microarray data

In the current study, the gene expression profiling data set (GSE33630 and GSE65144) and gene methylation profiling data set (GSE51090) were obtained from Gene Expression Omnibus (GEO, https://www.ncbi.nlm.nih.gov/geo/). A total of 60 THCA and 45 normal specimens were obtained in GSE33630, while 12 THCA and 13 normal samples were obtained in GSE65144. Both of these expression microarrays used the platform GPL570 (Affymetrix Human Genome U133 plus 2.0 Array, Thermo Fisher scientific, USA). For the gene methylation profiling microarray, GSE51090 included a total of 83 primary thyroid tumor samples and 8 adjacent normal tissue samples. The platform of this methylation microarray was GPL8490 (Illumina HumanMethylation27 BeadChip).

### 2.2 Data acquirement and processing

The raw data of the gene expression profiling datasets for GSE33630 and GSE65144 were downloaded from GEO public repositories. Subsequently, data were normalized and analyzed using GeneSpring GX 11.5 (Agilent Technologies Pty Ltd). The threshold set for upregulated and downregulated genes was a fold change ≥1.5 and *P* ≤ .05. For the gene methylation profiling data set (GSE51090), we used GEO2R online software to analyze the raw submitter‐supplied data of microarrays and identify DMGs. GEO2R is an interactive web tool that allows users to compare different groups of samples in a GEO series to screen genes that are differentially expressed in experimental conditions. *P* < .05 and |*t*| > 2 were used as the cutoff criteria to find DMGs. Finally, hypomethylation/high‐expression genes and hypermethylation/low‐expression genes were selected using a Venn diagram.

### 2.3 Functional and pathway enrichment analysis

Gene ontology (GO) analysis, including the cellular component, molecular function, and biological process, and the Kyoto Encyclopedia of Genes and Genomes pathway enrichment analysis were conducted for the selected hypermethylation/low‐expression genes and hypomethylation/high‐expression genes by Search Tool for the Retrieval of Interacting Genes (STRING). The STRING (https://string-db.org/) database was not only used to construct the PPI network but also offered systematic and integrative functional annotation tools for investigators to unravel the biological meaning behind an extensive list of genes. *P* < 0.05 was regarded as statistical significance.

### 2.4 PPI network construction and module analysis

The functional PPI analysis is essential for interpreting the molecular mechanisms of key cellular activities in carcinogenesis. In this study, we used the STRING database to construct the PPI network of hypomethylation/high‐expression genes and hypermethylation/low‐expression genes, respectively. An interaction score of 0.4 was regarded as the cutoff criterion, and the PPI was visualized. Subsequently, the Molecular Complex detection in Cytoscape software was conducted to screen modules within the PPI network with Molecular Complex detection score >3 and number of nodes >4. The top 3 hub genes were selected by CytoHubba app in Cytoscape software.

### 2.5 Validation of the hub genes in the TCGA database

The Cancer Genome Atlas (TCGA) database has generated comprehensive, multidimensional maps of the key genomic changes in various types of cancers. MEXPRESS (http://mexpress.be/) is a data visualization tool designed for the easy visualization of TCGA expression, DNA methylation, and clinical data, as well as the relationships between them. To confirm our results, we used MEXPRESS to validate hypermethylation/low‐expression hub genes and hypomethylation/high‐expression hub genes in TCGA database.

## 3 RESULTS

### 3.1 Identification of aberrantly methylated and DEGs in THCA

**Figure 1:**
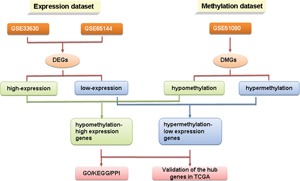
The flowchart of this study. DEG, differentially expressed gene; DMG, differentially methylated gene; GO, gene ontology; KEGG, Kyoto Encyclopedia of Genes and Genomes; PPI, protein‐protein interaction; TCGA, the Cancer Genome Atlas

**Figure 2:**
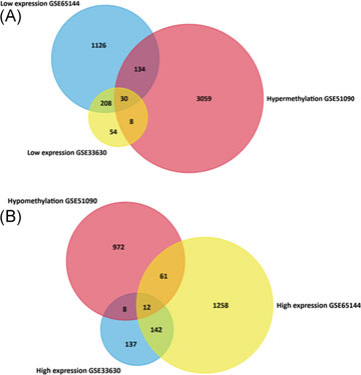
Identification of aberrantly methylated and differentially expressed genes was analyzed by Funrich software. Different color areas represented different datasets. (A) Hypermethylation and low expression genes. (B) Hypomethylation and high expression genes

**Figure 3:**
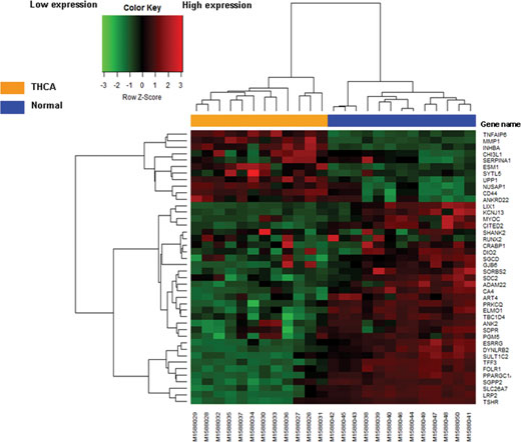
The heat map of 12 hypomethylation/high‐expression genes and 30 hypermethylation/low‐expression genes in GSE65144

The flowchart of this study is shown in Figure 1. For DEGs of the gene expression microarray, 154 overlapping upregulated genes (1473 in GSE65144 and 299 in GSE33630) and 238 overlapping downregulated genes (1498 in GSE65144 and 300 in GSE33630) were identified. For DMGs of the gene methylation microarray, 3231 hypermethylation genes and 1053 hypomethylation genes were found. Then, a total of 12 hypomethylation/high‐expression genes were obtained by overlapping 1053 hypomethylation genes and 154 upregulated genes; 30 hypermethylation/low‐expression genes were obtained by overlapping 3231 hypermethylation genes and 238 downregulated genes (Figure 2). The heat map of 12 hypomethylation/high‐expression genes and 30 hypermethylation/low‐expression genes in GSE65144 is shown in Figure 3.

### 3.2 GO and pathway functional enrichment analysis

**Figure 4:**
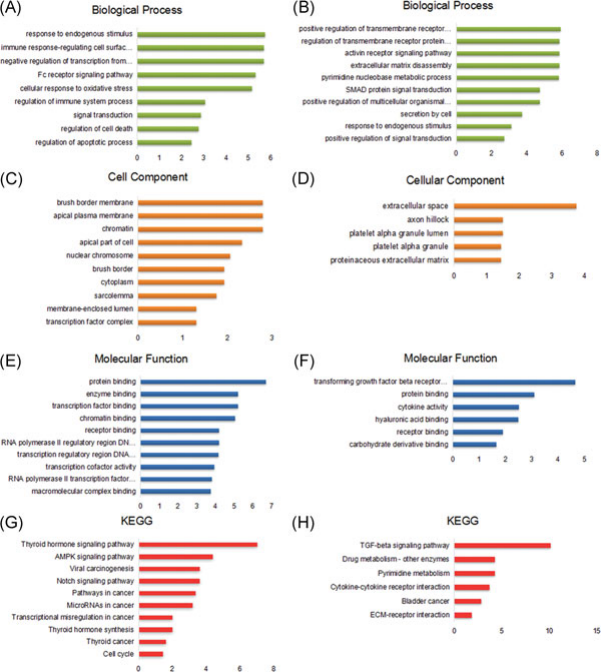
The gene ontology annotation and pathway enrichment analysis of all the aberrantly methylated and differentially expressed genes. (A) Biological process, (C) cellular component, (E) molecular function, and (G) KEGG of hypermethylation/low‐expression genes. (B) Biological process, (D) cellular component, (F) molecular function, and (H) KEGG of hypomethylation/high‐expression genes. The high enrichment score means that the genes were found more frequently in the particular ontology. KEGG, Kyoto Encyclopedia of Genes and Genomes

The GO annotation and pathway enrichment analysis of all the aberrantly methylated DEGs were implemented using the online tool STRING. Hypermethylation/low‐expression genes were enriched mainly in the response to hormone and endogenous stimulus (Figure 4A), and hypomethylation/high‐expression genes were mostly enriched in the regulation of transmembrane receptor protein, extracellular matrix disassembly, and activin receptor signaling pathway (Figure 4B). Cell component enrichment analysis indicated that hypermethylation/low‐expression genes were correlated with cytoplasm (Figure 4C), whereas hypomethylation/high‐expression genes were predominant at the extracellular region (Figure 4D). As for molecular function, hypermethylation/low‐expression genes were enriched mainly in protein binding, transcription factor binding, and enzyme binding (Figure 4E), while hypomethylation/high‐expression genes were mostly enriched in carbohydrate derivative binding, receptor binding, and hyaluronic acid binding (Figure 4F). The pathway analysis showed that hypermethylation/low‐expression genes were involved in the thyroid hormone signaling pathway, AMP‐activated protein kinase (AMPK) signaling pathway, viral carcinogenesis pathway, Notch signaling pathway, and pathway in THCA (Figure 4G), while hypomethylation/high‐expression genes significantly enriched in the transforming growth factor beta (TGF‐beta) signaling pathway, drug metabolism, and pyrimidine metabolism (Figure 4H).

### 3.3 PPI network construction, module analysis, and hub gene selection

**Figure 5:**
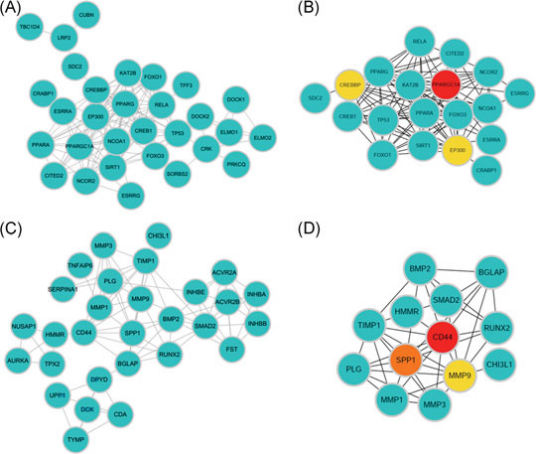
PPI network and hub genes of aberrantly methylated and differentially expressed genes. (A) PPI network and (B) hub genes for hypermethylation/low‐expression genes. (C) PPI network and (D) hub genes of hypomethylation/high‐expression genes. PPI, protein‐protein interaction

**Figure 6:**
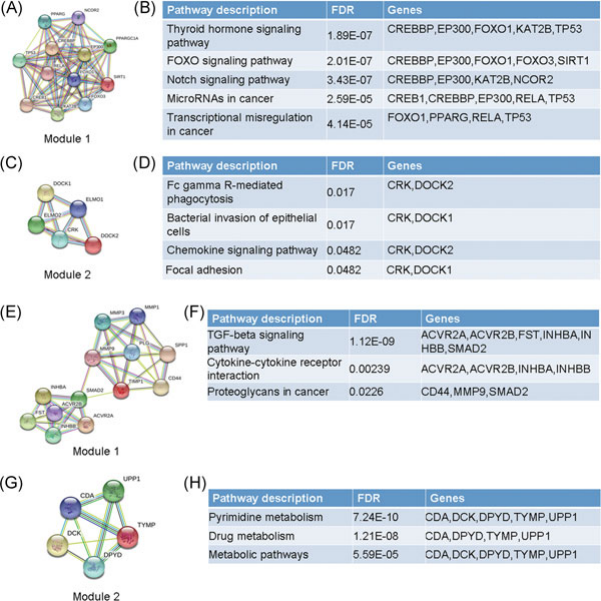
Top 2 modules for aberrantly methylated and differentially expressed genes. Hypermethylation/low‐expression genes: (A) module 1 and (B) the enrichment and pathway analysis of module 1. (C) Module 2 and (D) the enrichment and pathway analysis of module 2. Hypomethylation/high‐expression genes: (E) module 1 and (F) the enrichment and pathway analysis of module 1; (G) module 2 and (H) the enrichment and pathway analysis of module 2

PPI networks were constructed on the basis of the STRING database. Module analysis was conducted by Molecular Complex detection in Cytoscape software. Hub genes were selected by cytoHubba in Cytoscape software. For hypermethylation/low‐expression genes, the PPI network is shown in Figure 5A, and modules are displayed in Figure 6A,C. Significant core modules demonstrated functions of the thyroid hormone signaling pathway, FOXO signaling pathway, microRNAs in cancer, transcriptional misregulation in cancer, Fc gamma R‐mediated phagocytosis, and chemokine signaling pathway (Figure 6B,D). Top 3 hub genes were *PPARGC1A*, *CREBBP*, and *EP300* (Figure 5B). The PPI network of hypomethylation/high‐expression genes is illustrated in Figure 5C, and modules are displayed in Figure 6E,G. Significant vital modules showed functions including the TGF‐beta signaling pathway, cytokine‐cytokine receptor interaction, proteoglycans in cancer, pyrimidine metabolism, drug metabolism, and metabolic pathways (Figure 6F,H). Top 3 hub genes were *CD44*, *SPP1*, and *MMP9* (Figure 6D).

### 3.4 Validation of the hub genes in the TCGA database

**Figure 7:**
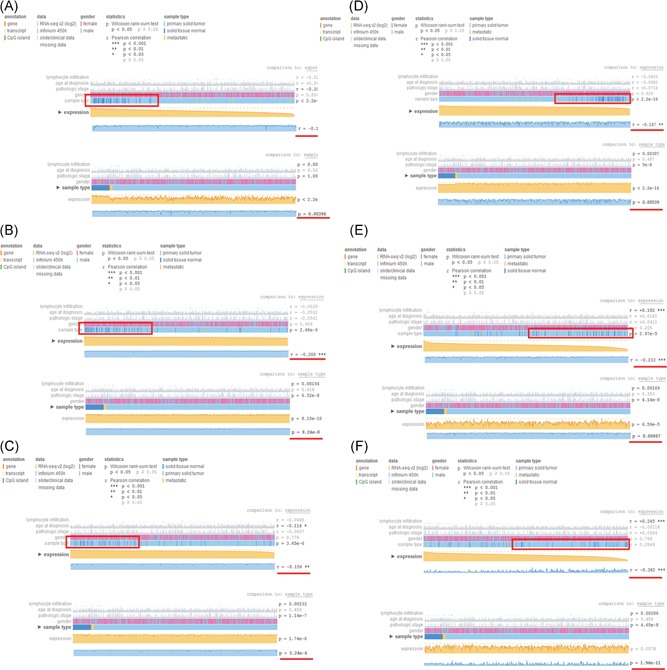
Validation of the hub genes in the TCGA database. In the default MEXPRESS plot, the samples are ordered by their expression value. These views show how hub gene expression and methylation are negatively correlated, which are confirmed by the Pearson correlation coefficients on the right. (A‐C) For the hypermethylation/low‐expression hub genes, normal samples tended to have higher expression than tumor samples. (D‐F) However, tumor samples tended to have higher expression than normal samples for hypomethylation/high‐expression hub genes. TCGA, the Cancer Genome Atlas

Hypermethylation/low‐expression hub genes and hypomethylation/high‐expression hub genes were then validated in another database TCGA to confirm the results. MEXPRESS is an intuitive web tool for the fast and straightforward querying and visualization of the relationship between expression and methylation in TCGA on a single‐gene level.^23^ In the default MEXPRESS plot, the samples are ordered by their expression value. These views show how hub gene expression and methylation are negatively correlated, which was confirmed by the Pearson correlation coefficients on the right of Figure 7. For the hypermethylation/low‐expression hub genes, normal samples tended to have higher expression than tumor samples (Figure 7A‐C). However, tumor samples tended to have higher expression than normal samples for hypomethylation/high‐expression hub genes (Figure 7D‐F). The outcomes are summarized in Table 1. The methylation and expression status was significantly altered and the same with our results, which suggested the stability and reliability of our findings.

**Table 1:** Validation of the hub genes in the TCGA database

Hub gene	Methylation status	*P* value	Expression status	*P* value
Hypermethylation/low‐expression				
PPARGC1A	Hypermethylation	.0039	Low expression	2.2 × 10^−16^
CREBBP	Hypermethylation	9.24 × 10^−8^	Low expression	8.15 × 10^−10^
EP300	Hypermethylation	3.24 × 10^−6^	Low expression	1.74 × 10^−6^
Hypormethylation/high‐expression				
CD44	Hypomethylation	.0054	High expression	2.2 × 10^−16^
SPP1	Hypomethylation	.0087	High expression	6.59 × 10^−5^
MMP9	Hypomethylation	1.96 × 10^−11^	High expression	0.058

TCGA, the Cancer Genome Atlas.

## 4 DISCUSSION

Exploring the underlying mechanisms of the initiation and development of THCA not only has prognostic implications but also may be helpful in monitoring the treatment response, surveillance for tumor recurrence, and guidance of clinical decisions. In recent years, microarrays based on high‐throughput platforms have emerged as a promising and efficient tool to screen the expression and methylation levels of thousands of genes simultaneously in the human genome. To our knowledge, the current study is the first to collectively analyze the information of both gene expression profiling microarrays and gene methylation profiling microarrays in the development of THCA.

The GO enrichment analysis revealed that the primary molecular functions of the hypermethylation/low‐expression genes were in the response to hormone and endogenous stimuli, while the hypomethylation/high‐expression genes were enriched mainly in the regulation of transmembrane receptor proteins, extracellular matrix disassembly, and the activin receptor signaling pathway. This finding is consistent with the knowledge that endogenous hormones play a significant role in THCA initiation and progression.^24^ The destruction of the extracellular matrix by enzymes often involves in tumor invasion and metastasis. Activin functions as a tumor‐suppressor protein and potently inhibits the growth of primary cultures of human follicular epithelial thyroid cells.^25^ Transmembrane receptors, such as G protein‐coupled receptor 30, play a fundamental role in cell proliferation, invasion, and metastasis in THCA.^26^ The Kyoto Encyclopedia of Genes and Genomes enrichment analysis of hypermethylation/low‐expression genes showed that methylation may affect the development and progression of THCA through the cancer‐associated pathways and AMPK signaling pathway. The AMPK pathway regulates both iodide and glucose uptake in normal thyroid cells, and it is highly activated in papillary thyroid carcinomas.^27^ The Kyoto Encyclopedia of Genes and Genomes pathway analysis of hypomethylation/high‐expression genes revealed that hypomethylated genes were involved in the pyrimidine metabolism and TGF‐β signaling pathway. Pyrimidine synthesis is vital for DNA replication in tumor cells,^28^ and TGF‐β acts as a tumor suppressor to impede progress of the cell cycle.^29^ Together, these results suggest that hypermethylation and hypomethylation play a critical role in cancer development.

The PPI network of hypermethylation/low‐expression genes illustrates an overview of their functional connections, of which, the top 3 hub genes were selected: *PPARGC1A*, *CREBBP*, and *EP300*. PPARG coactivator 1 alpha (PPARGC1A) functions as a mediator of the transcriptional outputs triggered by metabolic sensors, together orchestrating a network controlling cellular energy expenditure. Increased PPARGC1A expression might be an underlying feature of metastatic cancer progression.^30^ The tumor‐suppressor gene *EP300* plays an essential role in cell proliferation and differentiation. Somatic mutations of *EP300* are implicated in different types of cancer, including breast and ovarian cancers and cancer cell lines.^31^ CREB binding protein (CREBBP) is involved in the regulation of the cell cycle during the G1/S transition. Therefore, *PPARGC1A*, *CREBBP*, and *EP300* might be candidate genes for aberrant methylation that modulate energy metabolism, cell cycle, and proliferation in THCA. After constructing the PPI network for hypomethylation/high‐expression genes, the top 3 hub genes were *CD44*, *SPP1*, and *MMP9*. CD44 is a glycosylated transmembrane glycoprotein that plays a role in sustaining proliferation of THCA cells. The high expression of CD44 is a potential predictor of poor prognosis.^32^, ^33^ Secreted phosphoprotein 1 (SPP1) is a matricellular glycoprotein whose expression is elevated in various types of cancer and has been shown to be involved in tumorigenesis and metastasis in THCA.^34^ The function of matrix metalloproteinases (MMPs) is not only in the degradation of the extracellular matrix but also in development, angiogenesis, inflammation, cancer progression, and especially in promoting migration and invasion of cancer cells.^35^ In summary, these 3 genes are related to prognosis, tumorigenesis, and metastasis of THCA.

The top 2 modules of the PPI network of hypermethylation/low‐expression genes were associated with the thyroid hormone signaling pathway and Fc gamma R‐mediated phagocytosis, suggesting that the hypermethylation mainly affects the expression of genes in those pathways. The thyroid hormone signaling pathway is related to THCA initiation and progression. Fc gamma R‐mediated phagocytosis is responsible for removing the target tumor cells. Core modules within the PPI network of hypomethylation/high‐expression genes possessed functions including the TGF‐beta signaling pathway and pyrimidine metabolism, which affects DNA replication and the cell cycle in tumor cells.^28^

Previous studies using profiling arrays have mostly analyzed either methylation or gene expression data, but not both. Furthermore, individual investigations have limited numbers of overlapping gene profiles and insufficient power to identify critical genes and pathways. Our research jointly analyzed information on both gene expression profiling microarrays and gene methylation profiling microarrays by bioinformatics analysis of available microarray data. In this way, it is possible to come up with more reliable and precise screening results. However, we validated only candidate aberrantly methylated DEGs in the TCGA database. Further molecular biological experiments are needed to confirm the function of the identified genes in THCA.

In summary, our study provides a comprehensive bioinformatics analysis of aberrantly methylated DEGs that may be involved in the progression and development of THCA. In addition, we found 6 mostly changed hub genes including *PPARGC1A*, *CREBBP*, *EP300*, *CD44*, *SPP1,* and *MMP9*, which were significantly enriched in several pathways, including those associated with the thyroid hormone signaling pathway, AMPK signaling pathway, and the cell cycle in THCA. These findings may provide novel insights for unraveling the pathogenesis of THCA, and these candidate genes could serve as aberrant methylation‐based biomarkers for the precise diagnosis and treatment of THCA.

## CONFLICTS OF INTEREST

The authors declare that they have no conflict of interests

## AUTHOR CONTRIBUTIONS

WK and XC conceived and designed the study strategy. YQT and GRF statistically analyzed and interpreted the data. YQT, GRF, and HLX drafted or revisioned the manuscript. TSZ and HYS referenced collection and data management. YQT wrote the manuscript. GRF prepared the tables and figures. WK and XC supervised the study. All authors reviewed the manuscript.
